# Bis[methyl 3-(propyl­amino)­but-2-eno­ato]zinc

**DOI:** 10.1107/S160053681104520X

**Published:** 2011-11-05

**Authors:** Olamide O. Onakoya, Keneshia O. Johnson, Raymond J. Butcher, Jason S. Matthews

**Affiliations:** a Howard University, Department of Chemistry, 525 College Street N.W., Washington, DC 20059, USA

## Abstract

The title compound, [Zn(C_8_H_14_NO_2_)_2_], represents a zinc complex with the Zn^2+^ cation coordinated by two O and two N atoms in a distorted tetrahedral geometry.

## Related literature

For background to ZnO and its applications, see: Norton *et al.* (2004 ▶); Groenen *et al.* (2005 ▶); Wan *et al.* (2004 ▶). For the growth of ZnO, see: Tribolate *et al.* (1999 ▶); Fan *et al.* (2005 ▶); El Hichou *et al.* (2004 ▶); Hoon *et al.* (2011 ▶); Jong *et al.* (2009 ▶); Malandrino *et al.* (2005 ▶). For ZnO precursors, see: Smith (1983 ▶); Sato *et al.* (1994 ▶). The corresponding complex is a monomer; its structure consists of a Zn^2+^ cation with a distorted tetrahedral coordin­ation (Matthews *et al.*, 2006 ▶).
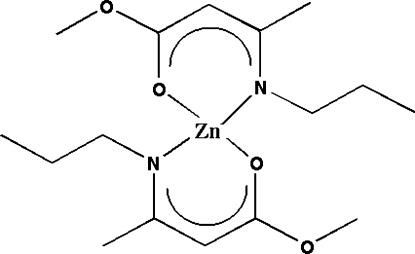


## Experimental

### 

#### Crystal data

[Zn(C_8_H_14_NO_2_)_2_]
*M* *_r_* = 377.77
Triclinic, 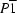

*a* = 7.8087 (5) Å
*b* = 9.4353 (6) Å
*c* = 12.8788 (11) Å
α = 76.820 (3)°
β = 77.381 (3)°
γ = 83.413 (3)°
*V* = 899.46 (11) Å^3^
*Z* = 2
Mo *K*α radiation
μ = 1.39 mm^−1^
*T* = 103 K
0.64 × 0.51 × 0.13 mm

#### Data collection

Bruker SMART CCD area-detector diffractometer
Absorption correction: multi-scan (*SADABS*; Sheldrick, 2002 ▶) *T*_min_ = 0.471, *T*_max_ = 0.840
9957 measured reflections
4977 independent reflections
4508 reflections with *I* > 2σ(*I*)
*R*_int_ = 0.020

#### Refinement

*R*[*F* ^2^ > 2σ(*F* ^2^)] = 0.027
*wR*(*F* ^2^) = 0.070
*S* = 1.00
4977 reflections
214 parameters
H-atom parameters constrained
Δρ_max_ = 0.82 e Å^−3^
Δρ_min_ = −0.54 e Å^−3^

### 

Data collection: *SMART* (Bruker, 1997 ▶); cell refinement: *SAINT* (Bruker, 1997 ▶); data reduction: *SAINT*; program(s) used to solve structure: *SHELXS97* (Sheldrick, 2008 ▶); program(s) used to refine structure: *SHELXL97* (Sheldrick, 2008 ▶); molecular graphics: *SHELXTL* (Sheldrick, 2008 ▶); software used to prepare material for publication: *SHELXTL*.

## Supplementary Material

Crystal structure: contains datablock(s) I, global. DOI: 10.1107/S160053681104520X/bt5685sup1.cif

Structure factors: contains datablock(s) I. DOI: 10.1107/S160053681104520X/bt5685Isup2.hkl

Additional supplementary materials:  crystallographic information; 3D view; checkCIF report

## Figures and Tables

**Table:** 

Zn—O1*B*	1.9784 (10)
Zn—N1*A*	1.9784 (12)
Zn—N1*B*	1.9785 (11)
Zn—O1*A*	1.9963 (10)

**Table:** 

O1*B*—Zn—N1*A*	117.85 (4)
O1*B*—Zn—N1*B*	97.63 (4)
N1*A*—Zn—N1*B*	123.66 (5)
O1*B*—Zn—O1*A*	106.41 (4)
N1*A*—Zn—O1*A*	96.73 (5)
N1*B*—Zn—O1*A*	114.34 (5)

**Fig. 1.**
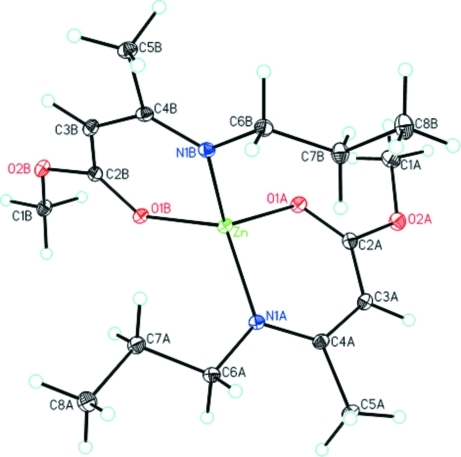
The molecular structure of the title compound, showing the atom-labeling scheme. Displacement ellipsoids are drawn at the 20% probability level and H atoms are shown as spheres of arbitrary radius.

## supplementary crystallographic information

### Comment

Novel precursors have been synthesized and utilized in the growth of ZnO thin films *via* metal-organic chemical vapor deposition (MOCVD). ZnO is a wide band gap (3.37ev) semiconductor, with several favorable properties including good transparency, high electron mobility, strong room-temperature luminescence and piezoelectric properties (Norton *et al.*, 2004). ZnO has a variety of potential applications such as gas sensors, ultraviolet light-emitting diodes, solar cells, photodetectors, transistors and laser systems (Groenen *et al.*, 2005) and (Wan *et al.*, 2004). These applications of ZnO have propelled researchers to develop methods for the growth of ZnO thin films. Techniques that have been employed include sublimation (Tribolate *et al.*, 1999), pulsed-laser deposition (PLD) (Fan *et al.*, 2005), spray pyrolysis (SP) (El Hichou *et al.*, 2004), magnetron sputtering (Hoon *et al.*, 2011) and MOCVD (Jong *et al.*, 2009). MOCVD has proven to be a promising method for ZnO growth due to a high degree of controllability of the film composition, capability for large scale area growth, high growth rate, prefered orientation and high quality thin films (Malandrino *et al.*, 2005). In order for the MOCVD process to produce uniform and reproducible films, the precursors employed need to be volatile and thermally stable. Previous studies have reported the use of metal alkyls such as diethyzinc in combination with an oxygen source (*e.g*.H_2_O or ROH) (Smith, 1983). The drawback with these precursors is that gas-phase pre-reaction occurs resulting in film contamination and precursor decomposition. In addition, dialkyzinc precursors of acetate, alkoxide and acetylacetonate have been employed (Sato *et al.*, 1994), however impurities are often found in prepared ZnO films. These drawbacks have sparked researchers interest in developing more favorable precursors for growing ZnO. Our research group has investigated the use of β-ketoiminate and β-iminoesterate ligand platforms for growing ZnO thin films (Matthews *et al.*, 2006). Herein we describe the synthesis, characterization, of a novel bis β-iminoesterate.The bond lengths and angles of the reported compound were compared to an analogous Zn bis β-iminoesterate complex that has been previously reported (Matthews *et al.*, 2006). The Zn—O bond lengths for the reported compound are longer than that observed for the analogous complex whose bond lengths measure 1.9454 Å and 1.9572 Å respectively. The Zn—N bond lengths are also longer in the analogous compound measuring 1.9475 Å and 1.9491 Å respectively. The is no difference between the Zn—O(1B) and Zn—N(1 A) bond lengths of 1.974 Å. However, Zn—O(1 A) and Zn—N(1B) measure 1.9963 Å and 1.9785 Å respectively.

### Experimental

Synthesis of bis [Methyl 3-*N*-(propylimino)butanoato] zinc (II) To a 100 ml round bottom flask Under an inert atmosphere of dry nitrogen, 2.00 g (12.7 mmol) of Methyl 3-*N*-(propylimino)butanoate was added to a Schlenk flask containing 50 ml of dried hexanes and a magnetic stir bar. The mixture was cooled to 0° and 6.4 ml s of diethyl zinc (1.0 *M*) was added drop wise by syringe. The mixture was allowed to warm up to room temperature and stirred for 1 h. The solvent was removed in vaccuo to afford a white solid. The isolated solid was dissolved in dry pentane and held at -5 °C for 2 days at which time the formation of colorless crystals was observed. Spectroscopic Analysis: ^1^H NMR 400 MHz, CDCl_3_, δ p.p.m.: 0.83 (t, 6H, C*H*_3_CH_2_CH_2_), 1.42 (m, 4H, CH_3_C*H*_2_CH_2_), 1.90 (s, 6H, C*H*_3_CN), 3.12 (m, 2H, CH_3_CH_2_C*H*), 3.20 (m, 2H, CH_3_CH_2_C*H*), 3.57 (s, 6H, OC*H*_3_), 4.28 (s, 2H, CC*H*CO); ^13^C NMR 100 MHz, CDCl_3_, δ p.p.m.: 11.70 [*C*H_3_CH_2_CH_2_], 22.19 [CH_3_*C*H_2_CH_2_], 24.63 [*C*H_3_CN], 50.89 [CH_3_CH_2_*C*H_2_], 52.30 [O*C*H_3_], 77.31 [*C*HCO], 171.54 [CH_3_*C*N], 172.31 [CH*C*O].

### Refinement

H atoms were positioned geometrically and refined using a riding model with C—H = 0.95 and 0.99 Å and with *U*_iso_(H) = 1.2 (1.5 for methyl groups) times *U*_eq_(C).

### Crystal data

**Table:**

[Zn(C_8_H_14_NO_2_)_2_]	*Z* = 2
*M**_r_* = 377.77	*F*(000) = 400
Triclinic, *P*1	*D*_x_ = 1.395 Mg m^−^^3^
Hall symbol: -P 1	Mo *K*α radiation, λ = 0.71073 Å
*a* = 7.8087 (5) Å	Cell parameters from 7001 reflections
*b* = 9.4353 (6) Å	θ = 2.3–24.6°
*c* = 12.8788 (11) Å	µ = 1.39 mm^−^^1^
α = 76.820 (3)°	*T* = 103 K
β = 77.381 (3)°	Plate, colourless
γ = 83.413 (3)°	0.64 × 0.51 × 0.13 mm
*V* = 899.46 (11) Å^3^	

### Data collection

**Table:**

Bruker SMART CCD area-detector diffractometer	4977 independent reflections
Radiation source: fine-focus sealed tube	4508 reflections with *I* > 2σ(*I*)
graphite	*R*_int_ = 0.020
phi and ω scans	θ_max_ = 30.7°, θ_min_ = 1.7°
Absorption correction: multi-scan (*SADABS*; Sheldrick, 2002)	*h* = −9→11
*T*_min_ = 0.471, *T*_max_ = 0.840	*k* = −12→12
9957 measured reflections	*l* = −17→18

### Refinement

**Table:**

Refinement on *F*^2^	Primary atom site location: structure-invariant direct methods
Least-squares matrix: full	Secondary atom site location: difference Fourier map
*R*[*F*^2^ > 2σ(*F*^2^)] = 0.027	Hydrogen site location: inferred from neighbouring sites
*wR*(*F*^2^) = 0.070	H-atom parameters constrained
*S* = 1.00	*w* = 1/[σ^2^(*F*_o_^2^) + (0.0336*P*)^2^ + 0.4769*P*] where *P* = (*F*_o_^2^ + 2*F*_c_^2^)/3
4977 reflections	(Δ/σ)_max_ = 0.003
214 parameters	Δρ_max_ = 0.82 e Å^−^^3^
0 restraints	Δρ_min_ = −0.54 e Å^−^^3^

### Special details

**Table:**

Geometry. All e.s.d.'s (except the e.s.d. in the dihedral angle between two l.s. planes) are estimated using the full covariance matrix. The cell e.s.d.'s are taken into account individually in the estimation of e.s.d.'s in distances, angles and torsion angles; correlations between e.s.d.'s in cell parameters are only used when they are defined by crystal symmetry. An approximate (isotropic) treatment of cell e.s.d.'s is used for estimating e.s.d.'s involving l.s. planes.
Refinement. Refinement of *F*^2^ against ALL reflections. The weighted *R*-factor *wR* and goodness of fit *S* are based on *F*^2^, conventional *R*-factors *R* are based on *F*, with *F* set to zero for negative *F*^2^. The threshold expression of *F*^2^ > σ(*F*^2^) is used only for calculating *R*-factors(gt) *etc*. and is not relevant to the choice of reflections for refinement. *R*-factors based on *F*^2^ are statistically about twice as large as those based on *F*, and *R*- factors based on ALL data will be even larger.

### Fractional atomic coordinates and isotropic or equivalent isotropic displacement parameters (Å^2^)

**Table:**

	*x*	*y*	*z*	*U*_iso_*/*U*_eq_	
Zn	0.54308 (2)	0.924864 (17)	0.751108 (12)	0.01951 (5)	
O1A	0.32476 (13)	0.81656 (11)	0.79509 (8)	0.0236 (2)	
O2A	0.15956 (15)	0.64045 (12)	0.90620 (9)	0.0302 (2)	
O1B	0.46956 (13)	1.13117 (11)	0.69599 (8)	0.02141 (19)	
O2B	0.47457 (14)	1.33455 (11)	0.56379 (8)	0.0244 (2)	
N1A	0.61225 (15)	0.87359 (13)	0.89469 (9)	0.0202 (2)	
N1B	0.69909 (15)	0.88855 (13)	0.61512 (9)	0.0199 (2)	
C1A	0.0663 (2)	0.65066 (19)	0.82025 (14)	0.0326 (3)	
H1AA	−0.0258	0.5813	0.8439	0.049*	
H1AB	0.1483	0.6278	0.7560	0.049*	
H1AC	0.0127	0.7499	0.8021	0.049*	
C2A	0.30075 (18)	0.72305 (15)	0.88369 (11)	0.0225 (3)	
C3A	0.3992 (2)	0.69008 (16)	0.96517 (11)	0.0246 (3)	
H3AA	0.3658	0.6099	1.0233	0.030*	
C4A	0.54370 (19)	0.76365 (15)	0.97014 (11)	0.0212 (3)	
C5A	0.6216 (2)	0.70783 (17)	1.07104 (12)	0.0276 (3)	
H5AA	0.7470	0.6799	1.0496	0.041*	
H5AB	0.5609	0.6227	1.1160	0.041*	
H5AC	0.6073	0.7848	1.1127	0.041*	
C6A	0.75495 (18)	0.94490 (15)	0.91732 (11)	0.0217 (3)	
H6AA	0.8526	0.8712	0.9317	0.026*	
H6AB	0.7105	0.9852	0.9835	0.026*	
C7A	0.8239 (2)	1.06658 (16)	0.82344 (12)	0.0252 (3)	
H7AA	0.7286	1.1437	0.8120	0.030*	
H7AB	0.8621	1.0279	0.7561	0.030*	
C8A	0.9787 (2)	1.13208 (18)	0.84601 (13)	0.0303 (3)	
H8AA	1.0179	1.2130	0.7856	0.045*	
H8AB	1.0756	1.0571	0.8533	0.045*	
H8AC	0.9417	1.1682	0.9136	0.045*	
C1B	0.3431 (2)	1.40060 (16)	0.63758 (12)	0.0254 (3)	
H1BA	0.3152	1.5019	0.6030	0.038*	
H1BB	0.3871	1.3986	0.7036	0.038*	
H1BC	0.2367	1.3466	0.6567	0.038*	
C2B	0.53266 (17)	1.19383 (15)	0.59936 (11)	0.0198 (2)	
C3B	0.65608 (19)	1.13903 (16)	0.52004 (11)	0.0227 (3)	
H3BA	0.6949	1.2061	0.4539	0.027*	
C4B	0.72970 (17)	0.99394 (15)	0.52762 (11)	0.0203 (2)	
C5B	0.8511 (2)	0.96427 (17)	0.42470 (11)	0.0251 (3)	
H5BA	0.9607	0.9128	0.4425	0.038*	
H5BB	0.8773	1.0569	0.3735	0.038*	
H5BC	0.7938	0.9039	0.3915	0.038*	
C6B	0.78253 (19)	0.74229 (15)	0.60851 (12)	0.0239 (3)	
H6BA	0.9067	0.7392	0.6156	0.029*	
H6BB	0.7819	0.7233	0.5361	0.029*	
C7B	0.6909 (2)	0.62329 (16)	0.69560 (13)	0.0276 (3)	
H7BA	0.5660	0.6270	0.6899	0.033*	
H7BB	0.6946	0.6397	0.7683	0.033*	
C8B	0.7791 (2)	0.47334 (17)	0.68364 (15)	0.0345 (3)	
H8BA	0.7201	0.3983	0.7421	0.052*	
H8BB	0.9032	0.4702	0.6881	0.052*	
H8BC	0.7706	0.4552	0.6130	0.052*	

### Atomic displacement parameters (Å^2^)

**Table:**

	*U*^11^	*U*^22^	*U*^33^	*U*^12^	*U*^13^	*U*^23^
Zn	0.01943 (8)	0.02190 (8)	0.01611 (8)	−0.00083 (6)	−0.00332 (5)	−0.00224 (5)
O1A	0.0198 (5)	0.0271 (5)	0.0231 (5)	−0.0020 (4)	−0.0047 (4)	−0.0026 (4)
O2A	0.0254 (5)	0.0325 (6)	0.0328 (6)	−0.0100 (4)	−0.0064 (4)	−0.0023 (4)
O1B	0.0212 (5)	0.0235 (5)	0.0181 (4)	−0.0015 (4)	−0.0029 (4)	−0.0023 (4)
O2B	0.0245 (5)	0.0230 (5)	0.0217 (5)	0.0005 (4)	−0.0015 (4)	−0.0003 (4)
N1A	0.0203 (5)	0.0225 (5)	0.0181 (5)	−0.0012 (4)	−0.0037 (4)	−0.0048 (4)
N1B	0.0175 (5)	0.0237 (5)	0.0195 (5)	−0.0019 (4)	−0.0048 (4)	−0.0055 (4)
C1A	0.0245 (7)	0.0379 (8)	0.0389 (8)	−0.0046 (6)	−0.0085 (6)	−0.0120 (7)
C2A	0.0197 (6)	0.0222 (6)	0.0244 (6)	−0.0017 (5)	−0.0011 (5)	−0.0054 (5)
C3A	0.0273 (7)	0.0235 (6)	0.0212 (6)	−0.0043 (5)	−0.0034 (5)	−0.0013 (5)
C4A	0.0224 (6)	0.0223 (6)	0.0181 (6)	0.0019 (5)	−0.0033 (5)	−0.0048 (5)
C5A	0.0337 (8)	0.0294 (7)	0.0189 (6)	−0.0040 (6)	−0.0074 (5)	−0.0003 (5)
C6A	0.0203 (6)	0.0258 (6)	0.0196 (6)	−0.0016 (5)	−0.0046 (5)	−0.0055 (5)
C7A	0.0219 (7)	0.0296 (7)	0.0233 (6)	−0.0043 (5)	−0.0044 (5)	−0.0028 (5)
C8A	0.0225 (7)	0.0362 (8)	0.0323 (7)	−0.0074 (6)	−0.0047 (6)	−0.0054 (6)
C1B	0.0256 (7)	0.0230 (6)	0.0244 (6)	0.0011 (5)	−0.0022 (5)	−0.0026 (5)
C2B	0.0163 (6)	0.0224 (6)	0.0209 (6)	−0.0030 (5)	−0.0062 (5)	−0.0019 (5)
C3B	0.0205 (6)	0.0254 (6)	0.0196 (6)	−0.0036 (5)	−0.0019 (5)	−0.0005 (5)
C4B	0.0152 (6)	0.0285 (6)	0.0188 (6)	−0.0034 (5)	−0.0047 (4)	−0.0062 (5)
C5B	0.0227 (7)	0.0326 (7)	0.0196 (6)	−0.0028 (5)	−0.0016 (5)	−0.0065 (5)
C6B	0.0235 (7)	0.0249 (6)	0.0237 (6)	0.0002 (5)	−0.0038 (5)	−0.0076 (5)
C7B	0.0242 (7)	0.0234 (7)	0.0331 (7)	−0.0011 (5)	−0.0028 (6)	−0.0048 (6)
C8B	0.0321 (8)	0.0245 (7)	0.0447 (9)	0.0001 (6)	−0.0043 (7)	−0.0070 (6)

### Geometric parameters (Å, °)

**Table:**

Zn—O1B	1.9784 (10)	C6A—H6AB	0.9900
Zn—N1A	1.9784 (12)	C7A—C8A	1.527 (2)
Zn—N1B	1.9785 (11)	C7A—H7AA	0.9900
Zn—O1A	1.9963 (10)	C7A—H7AB	0.9900
O1A—C2A	1.2653 (17)	C8A—H8AA	0.9800
O2A—C2A	1.3644 (17)	C8A—H8AB	0.9800
O2A—C1A	1.432 (2)	C8A—H8AC	0.9800
O1B—C2B	1.2666 (16)	C1B—H1BA	0.9800
O2B—C2B	1.3615 (16)	C1B—H1BB	0.9800
O2B—C1B	1.4292 (17)	C1B—H1BC	0.9800
N1A—C4A	1.3218 (18)	C2B—C3B	1.3915 (19)
N1A—C6A	1.4773 (18)	C3B—C4B	1.413 (2)
N1B—C4B	1.3197 (18)	C3B—H3BA	0.9500
N1B—C6B	1.4694 (18)	C4B—C5B	1.5143 (19)
C1A—H1AA	0.9800	C5B—H5BA	0.9800
C1A—H1AB	0.9800	C5B—H5BB	0.9800
C1A—H1AC	0.9800	C5B—H5BC	0.9800
C2A—C3A	1.392 (2)	C6B—C7B	1.513 (2)
C3A—C4A	1.411 (2)	C6B—H6BA	0.9900
C3A—H3AA	0.9500	C6B—H6BB	0.9900
C4A—C5A	1.515 (2)	C7B—C8B	1.526 (2)
C5A—H5AA	0.9800	C7B—H7BA	0.9900
C5A—H5AB	0.9800	C7B—H7BB	0.9900
C5A—H5AC	0.9800	C8B—H8BA	0.9800
C6A—C7A	1.515 (2)	C8B—H8BB	0.9800
C6A—H6AA	0.9900	C8B—H8BC	0.9800
			
O1B—Zn—N1A	117.85 (4)	C8A—C7A—H7AB	109.5
O1B—Zn—N1B	97.63 (4)	H7AA—C7A—H7AB	108.0
N1A—Zn—N1B	123.66 (5)	C7A—C8A—H8AA	109.5
O1B—Zn—O1A	106.41 (4)	C7A—C8A—H8AB	109.5
N1A—Zn—O1A	96.73 (5)	H8AA—C8A—H8AB	109.5
N1B—Zn—O1A	114.34 (5)	C7A—C8A—H8AC	109.5
C2A—O1A—Zn	119.24 (9)	H8AA—C8A—H8AC	109.5
C2A—O2A—C1A	116.87 (12)	H8AB—C8A—H8AC	109.5
C2B—O1B—Zn	120.03 (9)	O2B—C1B—H1BA	109.5
C2B—O2B—C1B	117.56 (11)	O2B—C1B—H1BB	109.5
C4A—N1A—C6A	117.53 (12)	H1BA—C1B—H1BB	109.5
C4A—N1A—Zn	120.54 (10)	O2B—C1B—H1BC	109.5
C6A—N1A—Zn	121.60 (9)	H1BA—C1B—H1BC	109.5
C4B—N1B—C6B	118.11 (11)	H1BB—C1B—H1BC	109.5
C4B—N1B—Zn	121.21 (9)	O1B—C2B—O2B	118.07 (12)
C6B—N1B—Zn	120.67 (9)	O1B—C2B—C3B	129.19 (13)
O2A—C1A—H1AA	109.5	O2B—C2B—C3B	112.75 (12)
O2A—C1A—H1AB	109.5	C2B—C3B—C4B	126.74 (13)
H1AA—C1A—H1AB	109.5	C2B—C3B—H3BA	116.6
O2A—C1A—H1AC	109.5	C4B—C3B—H3BA	116.6
H1AA—C1A—H1AC	109.5	N1B—C4B—C3B	124.95 (12)
H1AB—C1A—H1AC	109.5	N1B—C4B—C5B	120.39 (12)
O1A—C2A—O2A	117.82 (13)	C3B—C4B—C5B	114.65 (12)
O1A—C2A—C3A	129.20 (13)	C4B—C5B—H5BA	109.5
O2A—C2A—C3A	112.99 (12)	C4B—C5B—H5BB	109.5
C2A—C3A—C4A	126.59 (13)	H5BA—C5B—H5BB	109.5
C2A—C3A—H3AA	116.7	C4B—C5B—H5BC	109.5
C4A—C3A—H3AA	116.7	H5BA—C5B—H5BC	109.5
N1A—C4A—C3A	124.94 (13)	H5BB—C5B—H5BC	109.5
N1A—C4A—C5A	119.84 (13)	N1B—C6B—C7B	112.72 (11)
C3A—C4A—C5A	115.22 (12)	N1B—C6B—H6BA	109.0
C4A—C5A—H5AA	109.5	C7B—C6B—H6BA	109.0
C4A—C5A—H5AB	109.5	N1B—C6B—H6BB	109.0
H5AA—C5A—H5AB	109.5	C7B—C6B—H6BB	109.0
C4A—C5A—H5AC	109.5	H6BA—C6B—H6BB	107.8
H5AA—C5A—H5AC	109.5	C6B—C7B—C8B	110.83 (13)
H5AB—C5A—H5AC	109.5	C6B—C7B—H7BA	109.5
N1A—C6A—C7A	112.03 (11)	C8B—C7B—H7BA	109.5
N1A—C6A—H6AA	109.2	C6B—C7B—H7BB	109.5
C7A—C6A—H6AA	109.2	C8B—C7B—H7BB	109.5
N1A—C6A—H6AB	109.2	H7BA—C7B—H7BB	108.1
C7A—C6A—H6AB	109.2	C7B—C8B—H8BA	109.5
H6AA—C6A—H6AB	107.9	C7B—C8B—H8BB	109.5
C6A—C7A—C8A	110.95 (12)	H8BA—C8B—H8BB	109.5
C6A—C7A—H7AA	109.5	C7B—C8B—H8BC	109.5
C8A—C7A—H7AA	109.5	H8BA—C8B—H8BC	109.5
C6A—C7A—H7AB	109.5	H8BB—C8B—H8BC	109.5
			
O1B—Zn—O1A—C2A	−136.62 (10)	C6A—N1A—C4A—C3A	175.45 (13)
N1A—Zn—O1A—C2A	−14.94 (11)	Zn—N1A—C4A—C3A	−11.12 (19)
N1B—Zn—O1A—C2A	116.82 (10)	C6A—N1A—C4A—C5A	−4.78 (18)
N1A—Zn—O1B—C2B	132.15 (10)	Zn—N1A—C4A—C5A	168.66 (10)
N1B—Zn—O1B—C2B	−2.54 (11)	C2A—C3A—C4A—N1A	−2.6 (2)
O1A—Zn—O1B—C2B	−120.76 (10)	C2A—C3A—C4A—C5A	177.56 (14)
O1B—Zn—N1A—C4A	129.69 (10)	C4A—N1A—C6A—C7A	178.77 (12)
N1B—Zn—N1A—C4A	−108.15 (11)	Zn—N1A—C6A—C7A	5.41 (15)
O1A—Zn—N1A—C4A	17.10 (11)	N1A—C6A—C7A—C8A	−176.50 (12)
O1B—Zn—N1A—C6A	−57.14 (11)	Zn—O1B—C2B—O2B	178.12 (9)
N1B—Zn—N1A—C6A	65.01 (11)	Zn—O1B—C2B—C3B	−1.2 (2)
O1A—Zn—N1A—C6A	−169.74 (10)	C1B—O2B—C2B—O1B	−1.40 (18)
O1B—Zn—N1B—C4B	3.50 (11)	C1B—O2B—C2B—C3B	178.06 (12)
N1A—Zn—N1B—C4B	−127.45 (10)	O1B—C2B—C3B—C4B	5.5 (3)
O1A—Zn—N1B—C4B	115.43 (10)	O2B—C2B—C3B—C4B	−173.93 (13)
O1B—Zn—N1B—C6B	−175.72 (10)	C6B—N1B—C4B—C3B	178.33 (13)
N1A—Zn—N1B—C6B	53.32 (12)	Zn—N1B—C4B—C3B	−0.91 (19)
O1A—Zn—N1B—C6B	−63.79 (11)	C6B—N1B—C4B—C5B	−1.41 (19)
Zn—O1A—C2A—O2A	−173.49 (9)	Zn—N1B—C4B—C5B	179.35 (10)
Zn—O1A—C2A—C3A	6.7 (2)	C2B—C3B—C4B—N1B	−4.1 (2)
C1A—O2A—C2A—O1A	9.44 (19)	C2B—C3B—C4B—C5B	175.68 (14)
C1A—O2A—C2A—C3A	−170.72 (13)	C4B—N1B—C6B—C7B	−159.60 (13)
O1A—C2A—C3A—C4A	5.2 (3)	Zn—N1B—C6B—C7B	19.65 (16)
O2A—C2A—C3A—C4A	−174.58 (13)	N1B—C6B—C7B—C8B	178.50 (13)
